# Complete genome sequence of *Bulleidia* sp. 10714-15 isolated from human colon cancer patients

**DOI:** 10.1128/mra.00937-24

**Published:** 2024-10-29

**Authors:** Haruto Ikeda, Kota Oshibuchi, Jiayue Yang, Shinji Fukuda, Kazuharu Arakawa

**Affiliations:** ^1^Institute for Advanced Biosciences, Keio University, Tsuruoka, Yamagata, Japan; 2 Systems Biology Program, Graduate School of Media and Governance, Keio University, Fujisawa, Kanagawa, Japan; 3 MIRAI Technology Institute, Shiseido Co., Ltd., Yokohama, Japan; 4 Transborder Medical Research Center, Institute of Medicine, University of Tsukuba, Tsukuba, Ibaraki, Japan; 5 Gut Environmental Design Group, Kanagawa Institute of Industrial Science and Technology, Kawasaki, Kanagawa, Japan; 6 Laboratory for Regenerative Microbiology, Juntendo University Graduate School of Medicine, Bunkyo-ku, Tokyo, Japan; 7 Faculty of Environment and Information Studies, Keio University, Fujisawa, Kanagawa, Japan; University of Maryland School of Medicine, Baltimore, Maryland, USA

**Keywords:** *Bulleidia* sp., Nanopore sequencing, complete genome, colon cancer

## Abstract

*Bulleidia* sp. is a non-spore-forming, obligatory anaerobic, Gram-positive bacterium isolated from the stool samples of human colon cancer patients. We report the complete genome sequence of *Bulleidia* sp. 10714-15, comprising a single linear chromosome of 2,218,984 bp with a G + C content of 36.6%.

## ANNOUNCEMENT

Erysipelotrichidae are non-spore-forming, Gram-positive bacteria often isolated from stool samples. Bacteria in this group, such as *Solobacterium moorei,* are suggested to promote the progression of colon inflammation (1). We, therefore, isolated bacteria from the stool samples of human colon cancer patients and identified a bacterium belonging to this group but with lowered biofilm formation, which was later determined to be *Bulleidia* sp. based on GTDB Taxonomy Assignment (2) with the genome sequence (ANI = 96.67 compared to GCF_015256775.1, *Bulleidia* sp.). This bacterium could be useful to study its effects on colon inflammation as well as for comparative studies of Erysipelotrichidae for their biofilm formation abilities. To this end, here we report the complete genome sequence of *Bulleidia* sp.

*Bulleidia* sp. obtained from stool specimens of human colon cancer patients was cultured in GAM medium at 37°C under anaerobic conditions for 24 hours, both for isolation and sequencing. The genomic DNA was extracted from the harvested cells using NucleoBond HMW DNA (MACHEREY-NAGEL) according to the manufacturer’s protocol. A nanopore sequencing library was prepared using the Rapid Barcoding Sequencing Kit (SQK-RBK114) (Oxford Nanopore Technologies) and sequenced using an R10.4.1 Flowcell (FLO-MIN114) on a GridION X5 device (GridION software release 12 July 2023; Oxford Nanopore Technologies). Base calling (Super Accurate Mode) and demultiplexing were performed using Guppy version 6.5.7 software, resulting in 177,068 reads of *N*_50_ length 17,846 bp, totaling 1.577 Gbp. Adapters were removed with Porechop version 0.2.3 (3), and reads less than 50 kbp in length were filtered out (252 Mbp remaining, nearly 100× coverage) prior to assembly with Canu version 2.2 (4). Error correction was performed using medaka version 1.11.3 with Dorado version 0.7.0 corrected reads (5). Assembly quality was assessed using CheckM version 1.2.2 (6) with automatic lineage identification that resulted in k__Bacteria (UID2372) with 100% completeness. The genome was annotated using the DDBJ Fast Annotation and Submission Tool (DFAST) version 1.2.18 (7). All software was used with default settings unless otherwise specified.

The assembled genome consists of one linear chromosome of 2,128,984 bp with a GC content of 36.6%, harboring 2,045 protein-coding genes, 9 rRNAs, and 42 tRNAs. The availability of the genome resource can potentially contribute to the molecular mechanisms of the lowered biofilm formation in this species compared to other Erysipelotrichidae.

## Data Availability

The genome sequences reported here were deposited in DDBJ under accession number AP036017, and the raw reads were deposited in the Sequence Read Archive (SRA) under BioProject accession number PRJNA1144681 as SRR30220828 run.
